# Competitive oscillatory dynamics in excitable neuron networks

**DOI:** 10.3389/fnetp.2025.1613288

**Published:** 2025-09-05

**Authors:** Zhigang Zheng, Lin Yan, Tao Li, Jiajing Liu, Lei Wang, Yu Qian

**Affiliations:** ^1^ Institute of Systems Science, Huaqiao University, Xiamen, China; ^2^ College of Information Science and Engineering, Huaqiao University, Xiamen, China; ^3^ School of Mathematical Sciences, Huaqiao University, Quanzhou, China; ^4^ College of Physics and Optoelectronic Technology, Baoji University of Arts and Sciences, Baoji, China

**Keywords:** self-sustained oscillation, excitable neuron network, winfree loop, loop-loop competition, loop-hub competition

## Abstract

Collective dynamics of networks of excitable neurons can be considered as the emergence of ordering from microscopic self-organization at the macroscopic scale. Sustained oscillation can emerge on networks of neurons even if a single neuron is dynamical excitable and non-oscillatory. Fundamental ingredients of networks such as loops, trees, and hubs, play distinct roles in supporting, propagating and impeding sustained oscillations. In this paper, we explore the mechanism of collective self-sustained oscillations on neuron networks by analyzing the functions of different topologies in shaping the oscillatory patterns on excitable neuron networks. The Winfree loops are revealed to be responsible for generating collective oscillations as the oscillation core, and other neurons act as the propagating paths. The existence of large numbers of loops in a network indicates potential competitions of the formation of collective oscillatory dynamics. The roles of loop-loop competition in homogeneous networks and loop-hub competition in heterogeneous networks are extensively discussed.

## 1 Introduction

Human brain consists of billions of neurons and may exhibit diverse ongoing and stimulus-evoked electro-physiological activity patterns covering broad spatial and temporal scales (Bullmore and Sporns, 2009). It is significant to understand the origins and dynamical mechanisms underlying the complexity across various scales (Fries, 2005), which is crucial to unveil brain functions and behaviors (Singer, 1999). Practically, these efforts are beneficial to developing therapies for brain diseases, and designing brain-inspired intelligent systems (Mcintosh, 2000; Seth and Bayne, 2022).

Oscillation is a very ubiquitous and common phenomenon in nature (Winfree, 1967; Pikovsky et al., 2001), which occurs in physics, chemistry, and biology, such as human metabolism, signal propagation, and spatiotemporal pattern dynamics (Strogatz, 1994; Rodrigues et al., 2016). Various complex oscillatory activities in neuronal networks can be observed at different levels (Park and Friston, 2013). Microscopically, an individual neuron remains at its resting state in the absence of external pacings. In some cases, regular firing of neurons has also been observed, for example, in central patterns generators that produce rhythmic motor patterns such as breathing, walking and swimming are oscillators (Marder and Bucher, 2001; Marder et al., 2005). Clustered and partially synchronous spikes of neurons can be found at the mesoscopic level. At the macroscopic neural-network level, collective population activity manifests as oscillations with nested and broad-spectrum distributed frequencies (Rabinovich et al., 2006; Izhikevich, 2006).

Self-sustained oscillation in excitable media systems, especially neuron networks, is a rather ubiquitous dynamical phenomenon, and they are closely related to issues such as synchronization (Pikovsky et al., 2001; Zheng, 2019), nonlinear wave propagation (Zheng, 2004), pattern dynamics (Cross and Hohenberg, 1993; Cross and Greenside, 2009), and the dynamics of biological networks (Newman, 2003; Barabási and Oltvai, 2004; Arenas et al., 2008), where network topology plays an important role. The basic mechanisms of oscillatory activity (in different frequency bands) in the brain such as the interplay between excitation and inhibition have been extensively explored (Buzsáki and Draguhn, 2004; Tiesinga and Sejnowski, 2009; Wang, 2010).

Studies of self-sustained oscillatory dynamics are also beneficial to spatiotemporal pattern formations. In the 1950s, Turing discovered that activators and inhibitors with different diffusion coefficients can spontaneously transform reaction-diffusion systems into spatially periodic oscillatory non-uniform states (Turing, 1952). In the 1970s (Othmer and Scriven, 1971; 1974), found that Turing instability also plays an important role in systems with network structures (Cross and Greenside, 2009).

Feedback is an important mechanism responsible for the emergence of oscillatory behaviors on networks of non-oscillatory nodes. This mechanism was originated from extensive studies of biochemical oscillations discovered since 1960s, such as glycolysis reactions and horseradish peroxidase reactions (Steele et al., 2006). The mechanism of these behaviors have been unveiled by exploring the building blocks of self-organized oscillations in gene regulatory networks (Zhang et al., 2012; Zhang et al., 2014b; Zhang et al., 2014a).

Neural network is composed of a large number of neurons with synaptic connections. A single neuron is usually excitable while non-oscillatory, which means a neuron stays in its resting state in the absence of external stimuli. However, the emergence of self-sustained oscillations in coupled networks composed of non-oscillatory neurons is clearly a non-trivial collective behavior. For an autonomous network, a topological feedback mechanism is required for the emergence of the collective sustained oscillation. Since each neuron in the system does not exhibit oscillatory behavior on its own, the feedback mechanism for self-sustained oscillations should be due to the interactions between neurons. Winfree loop is a recurrent network topology that plays such a key role (Winfree, 1991).

The self-sustained oscillations on neural networks can be traced back to the 1970s, when Wilson and Cowan studied the structure of neural networks and the impact of memory patterns (Wilson and Cowan, 1972). Hopfield investigated the storage and retrieval of memory from the perspective of attractor dynamics (Hopfield, 1982; 1984). Gutkin et al. studied the switching phenomena of self-sustained oscillations in brain memory (Gutkin et al., 2001). With the rise of complex networks, an increasing number of researchers have begun to study the self-sustained oscillations in neural networks from the perspective of network dynamics, attracting widespread attention. Roxin *et al.* studied self-sustained oscillations based on small-world networks (Roxin et al., 2004). Tinsley *et al.* found that typical target wave patterns can emerge in small-world networks composed of excitable nodes (Tinsley et al., 2005).

The role of Winfree loops has not been well unveiled until the proposition of the dominant phase advanced driving approach (Qian et al., 2010). By using this methos, it was found that very complex spatiotemporal patterns, such as spiral wave patterns, target wave patterns, and patterns coexisting with spiral and target waves, can be obtained in small-world networks. Liao et al. (2011) found that in complex networks with random connections, the typical target wave pattern is a disordered self-sustained oscillatory pattern. It was further discovered that Winfree loops extensively exist in complex networks, which can maintain self-sustained oscillations (Qian et al., 2010; Zheng and Qian, 2018; Qian et al., 2019). We recently revealed that the minimum Winfree loop determines the self-sustained oscillation on excitable Erdos-Renyi random networks (Qian et al., 2017).

Since Winfree loop plays important roles in governing oscillatory behaviors on neuron networks, it is valuable to make clear some key issues of Winfree-loop dynamics. First, there exist numerous cycles on a network, all these cycles are potential candidates of Winfree loops. Therefore the understanding of the stability and the competition among different oscillatory modes becomes significant. Second, nodes on networks may exhibit highly heterogeneous feature, the influence of this property on the stability of Winfree loop needs to be extensively investigated. These issues will be our focus in this paper. We will extensively investigate the sustained oscillations on networks of excitable neurons by concentrating on the mechanism of the emergence of self-sustained oscillation on excitable neuron networks in the absence of external forcing.

In this paper, we explore the topological feedback mechanism of collective self-sustained oscillations on neuron networks by analyzing the functions of different topologies in shaping the oscillatory patterns on excitable neuron networks. The Winfree loops are revealed to be responsible for generating collective oscillations as the oscillation core, and other neurons act as the propagating paths. It is revealed that fundamental ingredients of networks, e.g., loops, trees, and hubs, play distinct roles in supporting, propagating and impeding sustained oscillations. The stability of sustained oscillation on different Winfree loops is studied by analyzing the impact of its neighboring loops and hub nodes. The existence of large numbers of loops in a network indicates potential competitions of the formation of collective oscillatory dynamics. Loop-hub competition implies that Winfree loops with low-degree nodes are more stable on heterogeneous networks. These fundamental results provide valuable knowledge on the working mechanism of possible rhythmic behaviors and the corresponding functions such as memory on neural networks.

## 2 Loop sustained oscillation

### 2.1 Loops on networks

As the emergent consequence, the topological structure of a network may significantly influence the collective dynamics. It is important to understand the basic building blocks of networks such as nodes and edges in dominating the self-organization process. Identifying key nodes and edges provides new mechanisms to understand the both the structural and dynamical behaviors of a network.

Although a lot of effort has been focused on the ingredients of networks, such as nodes and links, the most important building blocks that support collective dynamics are essentially communities, loops, or hubs, which provide dynamical feedback or dominance in governing global dynamics in networks (Newman, 2003). An L -loop is topologically defined as a closed self-avoiding path along L distinct vertices, where L is the loop length. There exist a large number of loops with different lengths L . Counting the number of loops is a challenging combinatorial problem despite of its topological simplicity (Noh, 2007; Marinari et al., 2007; Jiang et al., 2023).

Counting the number of loops and its statistics have been computed in various real networks. Let M(L,N) denote the number of L -loops in a network with N vertices. Bianconi and Capocci (2003) found that the BA scale-free network exhibits a logarithmic scaling M(L,N)∼(ln⁡N)L . Marinari et al. (2007) studied the loop statistics in k -homogeneous (namely, all vertices have the same degree k ) random networks and found that $M\left(L,N\right)={\left(k-1\right)}^{L}/\left(2L\right)$ (Kim and Kim, 2005).

Meanwhile, loop structure promotes network function in many ways. Node centrality defined by the cycle structure performs well in spreading and control processes (Fan et al., 2020). Cycles are the dominant contributors to information storage capability. Networks with cycle structure have optimal synchronizability (Zheng, 2019).

We are concerned with the role of loops in manipulating sustained oscillations as an emergent behavior on complex networks and its stability by considering the competitions among loops and other ingredients.

### 2.2 The Bär-Eiswirth model

We adopt the Bär-Eiswirth excitable model (Bär and Eiswirth, 1993) as the local neuronal dynamics. The evolution of the excitable network can be written by

$$\frac{\text{d}u_{i}}{\text{d}t}=\frac{1}{\varepsilon }u_{i}\left(1-u_{i}\right)\left(u_{i}-\frac{v_{i}+b}{a}\right)+\frac{D}{k_{i}}\overset{N}{\underset{j=1}{\sum }}A_{i,j}\left(u_{j}-u_{i}\right),$$ (1)

$$\frac{\text{d}v_{i}}{\text{d}t}=f\left(u_{i}\right)-v_{i}.$$ (2)

Here f(u) is a piecewise function and follows

$$f\left(u\right)=\left\{\begin{array}{cc} 0\; & u<\frac{1}{3},\\ 1-6.75u{\left(u-1\right)}^{2}\; & \frac{1}{3}\leq u\leq 1,\\ 1\; & u>1. \end{array}\right.$$

The nodes are labeled by subscripts i,j=1,2,…,N . Physically the excitable variables u and v are respectively the membrane potential and the recovery current in the case of imitating neural dynamics. The local excitable dynamics is governed by dimensionless parameters a , b and ε , among which parameters a and b determine the excitation threshold and ε regulates the time scale between u and v variables. An excitable node is non-oscillatory and in its rest state. Oscillation occurs if and only if an excitable node is persistently paced by an external stimulus that exceeds the excitation threshold.

The interactions between the nodes on the network are realized by the diffusion coupling term $\frac{D}{k_{i}}\sum_{j=1}^{N}A_{i,j}\left(u_{j}-u_{i}\right)$ , where A is the adjacency matrix and defined as $A_{i,j}=A_{j,i}=1$ if there is an edge connecting nodes i and j , and $A_{i,j}=A_{j,i}=0$ otherwise. The coupling strength D determines the interaction strength between a pair of connected nodes. $k_{i}$ symbolizes the degree of the i -th node.

Throughout this paper, we are concerned with the significance of topology of networks on collective sustained oscillations. It has been found that main conclusions obtained in the following discussions do not depend on detailed excitable node dynamics, e.g., the FitzHugh-Nagumo model, the Izhikevich model, or parameters for a specific model (Qian et al., 2017). One performs numerical simulations by integrating Equations 1, 2. Parameters of node dynamics are fixed as a=0.84 , b=0.07 , ε=0.04 , which correspond to excitable feature of a neuron. Variations of these parameters do not qualitatively affect the following results. The coupling strength is fixed as D=1.5 for most cases of the following discussions. Initial states of excitable nodes on the network $\left\{u_{i}\left(t=0\right),v_{i}\left(t=0\right)\right\}$ (i=1,2,…,N) are randomly chosen in the range [0,1].

### 2.3 Sustained oscillations on a loop

Let us first study the dynamics of a single-loop network. It should be emphasized that multiple dynamics can occur even on a simple loop network. Here we discuss the simplest case of single pulse on the loop.

**FIGURE 1:**
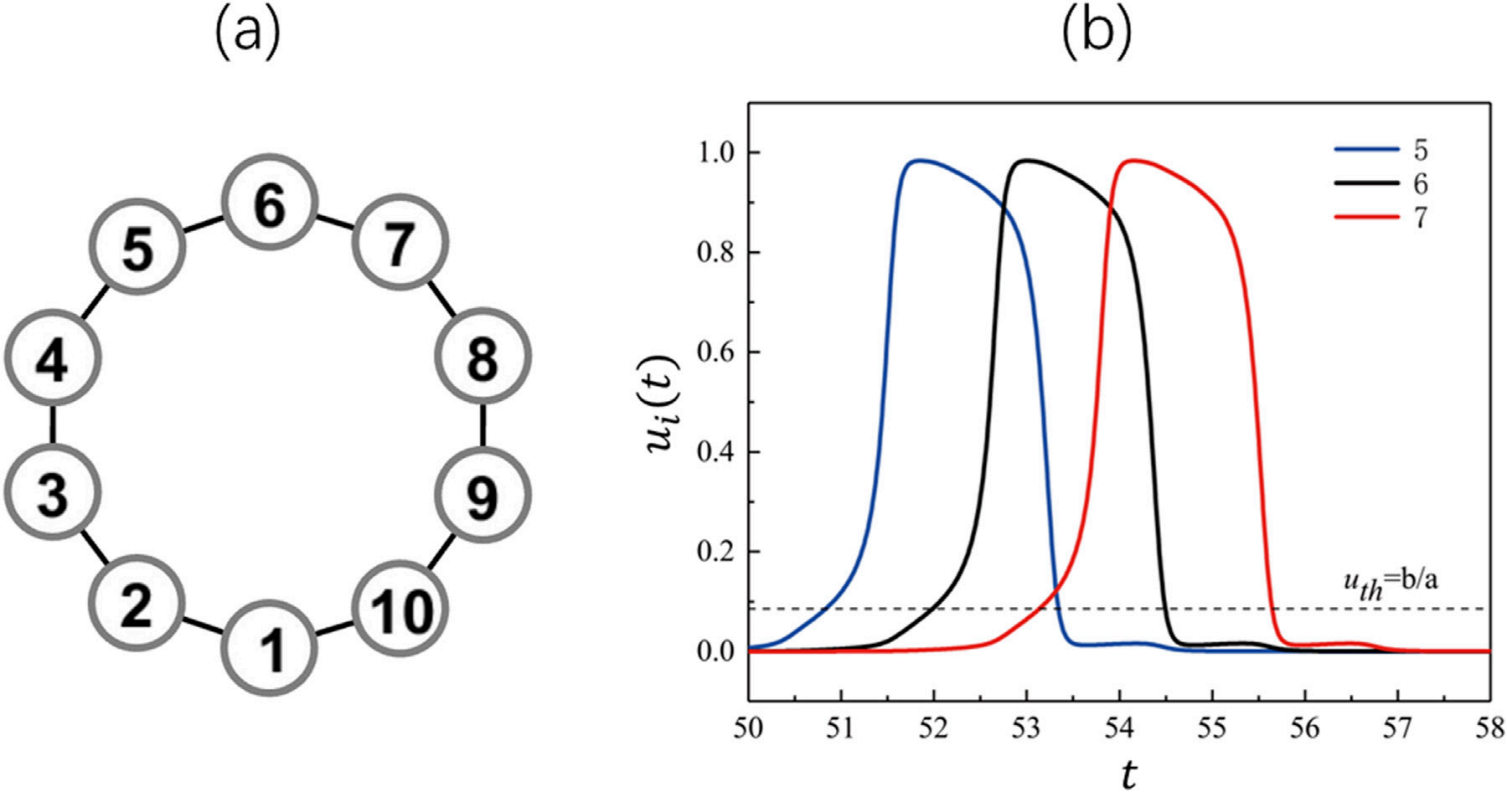
**(a)** A single loop network. **(b)** Sustained oscillation of nodes in the loop.

To accomplish a sustained oscillatory wave on the loop, for example, on the L=10 loop shown in Figure 1a, we first cut the loop into a chain by removing one edge, e.g., the edge $e_{1,10}$ . One excites node i=1 by a large stimulation exceeding its threshold, where a spiking pulse occurs at node i=1 , i.e., $u_{1}\left(t\right)$ exhibits a peak behavior. This peak then arouses its neighbor node i=2 to the spiking state. This excitation propagates sequentially along the chain. Before the spike moves to node i=10 , one recovers the edge $e_{1,10}$ to form again a loop. This leads to the recurrence of excitation of i=1 by i=10 , and henceforth a persistent oscillatory spiking wave runs along the loop.

Figure 1b presents the temporal behavior in one cycle of spike propagation on the loop by plotting the spiking behavior of nodes i=5,6,7 . It can be clearly found that these pulses occur with equal delay time interval τ , which depends on parameters a,b and coupling strength D . The period of the above sustained oscillation is determined by the length of the loop, i.e., T=Lτ (Zheng and Cross, 2003). For convenience, we call the oscillation produced by the loop L as the L *-mode oscillation*.

## 3 Multiple oscillatory patterns

### 3.1 Dominant phase-advanced driving (DPAD) method

To unveil the building blocks of the oscillation embedded on general neuron networks, a recently promising approach is called the *dominant phase-advanced driving* (DPAD) method (Qian et al., 2010; Liao et al., 2011; Zheng and Qian, 2018), which intends to find the strongest cross driving of the target node when a system is in an oscillatory state from the viewpoint of loop feedback. The basic idea of DPAD is a comparison and ordering of the significance of nodes in a network with sustained oscillations based on their phase dynamics.

**FIGURE 2:**
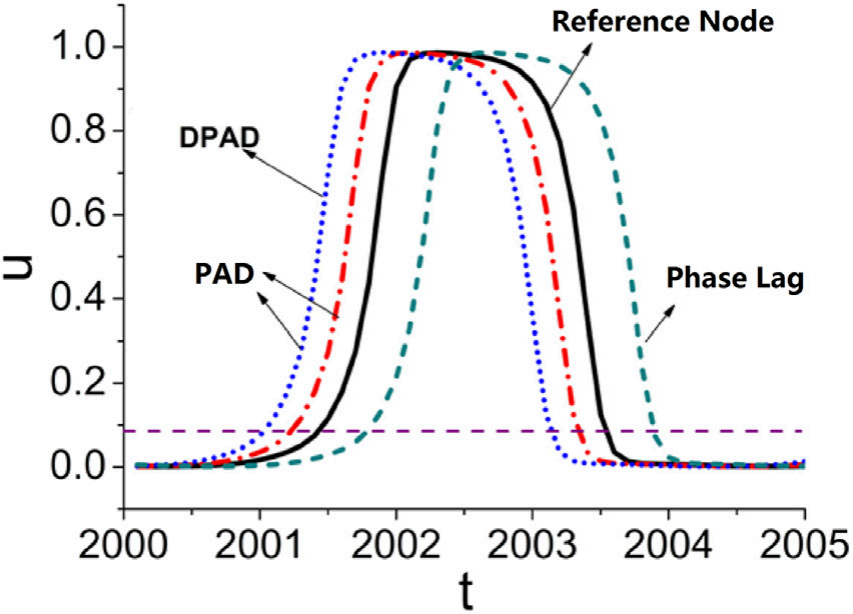
A schematic plot of the DPAD approach. The reference oscillatory time series is labeled as black solid line, the phase-lagged, PAD, and DPAD lines are labeled as green dashed, red dot-dashed, and blue dotted lines, respectively.

The identification of DPAD is schematically shown in Figure 2. The oscillatory behavior of an individually non-oscillatory node is apparently driven by signals from one or more interactions with advanced phases, called as the *phase-advanced driving* (see blue and red lines in Figure 2, also see the green *phase-lagged* line as a comparison.). Among all phase-advanced interactions, the interaction making the most significant contribution to the given node is defined as the dominant phase-advanced driving (DPAD) (see the blue line). By applying this reduction approach, the original oscillatory high-dimensional complex network of N nodes with M vertices/interactions can be simplified to a one-dimensional directed network of size N with $M^{\prime }<M$ directed dominant phase-advanced interactions.

### 3.2 Multiple oscillation patterns

**FIGURE 3:**
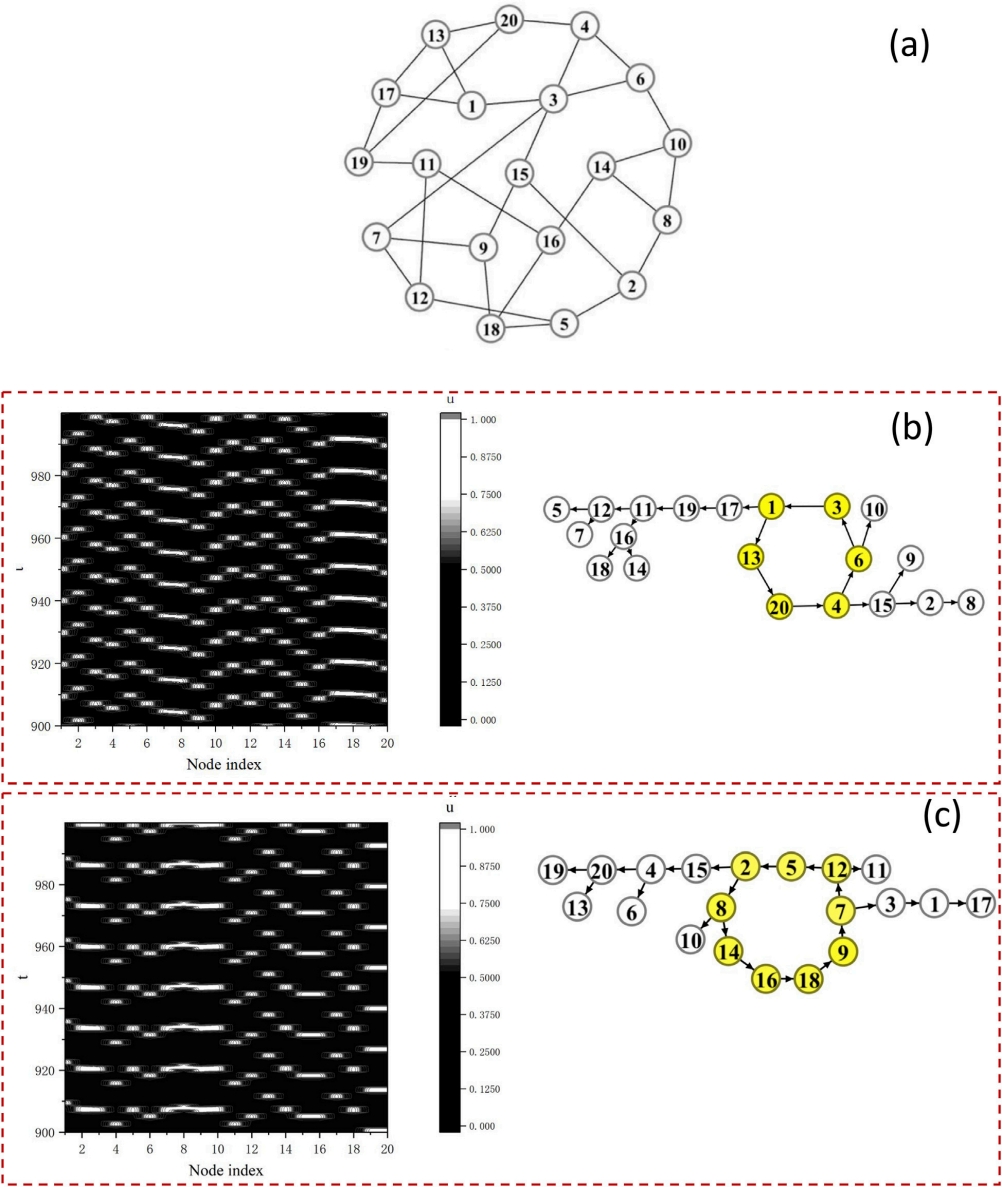
Miscellaneous oscillation patterns on a homogeneous random network starting from different initial conditions. **(a)** The homogeneous network with N=20 neurons and degree k=3 . **(b)** Spatiotemporal dynamics of the network by recording the membrane voltage $u_{i}\left(t\right)$ (left) and the DPAD graph of sustained oscillations composed of a L=5 loop shown by yellow nodes (right). **(c)** Spatiotemporal dynamics of $u_{i}\left(t\right)$ (left) and the DPAD graph with a L=9 loop (right).

Let us first explore various possible oscillatory patterns by starting from different initial states. We study this by using the homogeneous random network with N=20 Bär-Eiswirth neurons and degree k=3 , as shown in Figure 3a. Two typical distinct spatiotemporal patterns are given in the left panels of Figures 3b,c. Obviously these two patterns possess different spatial ordering and different temporal rhythms.

It is pertinent to identify the principal modes embedded in these different patterns. This can be accomplished by performing the DPAD approach. In right panels of Figures 3b,c, the reduced directed networks corresponding to the oscillatory dynamics by using the DPAD method are obtained. For the dynamics shown in left panel of Figure 3b, the DPAD network is composed of two types of topologies. First, a single L=6 Winfree loop $L_{6}=\left(1\to 13\to 20\to 4\to 6\to 3\to 1\right)$ plays the role of oscillation source, with cells in the loop exciting sequentially to maintain the self-sustained oscillation. Secondly, other neurons act as propagators of the sustained oscillation, namely, spike waves propagate downstream along several trees rooted at different neurons in the loop. This indicates that the DPAD structure well illustrates the wave propagation paths.

The dynamics presented in the left panel of Figure 3c is destructed by plotting the corresponding DPAD topology, as shown in the right panel of Figure 3c. A a longer (L=9) Winfree loop $L_{9}=\left(2\to 8\to 14\to 16\to 18\to 9\to 7\to 12\to 5\to 2\right)$ may support a longer-period oscillatory mode, and other neurons build multiple propagating paths with different lengths.

The above discussions indicate the distinctive significance of some units in the oscillation. Because the Winfree loop works as the oscillation source, units in the loop should be more important in the contributions of the oscillation.

## 4 Loop-loop competition

The above studies unveil that different oscillation patterns starting from different initial states are dominated by different source loops. The DPAD approach provides a powerful tool in excavating the dominant loop that governs the global oscillation on the network. On the other hand, there exists a large number of loops on a network as potential candidates of oscillation sources. Obviously the persistent oscillation from a loop depends on its dynamical stability. It is important to examine the stability of the oscillation generated by a given loop. The stability of a given loop oscillation depends on the competition with its neighbor loops.

### 4.1 Stability of short-loop oscillation

**FIGURE 4:**
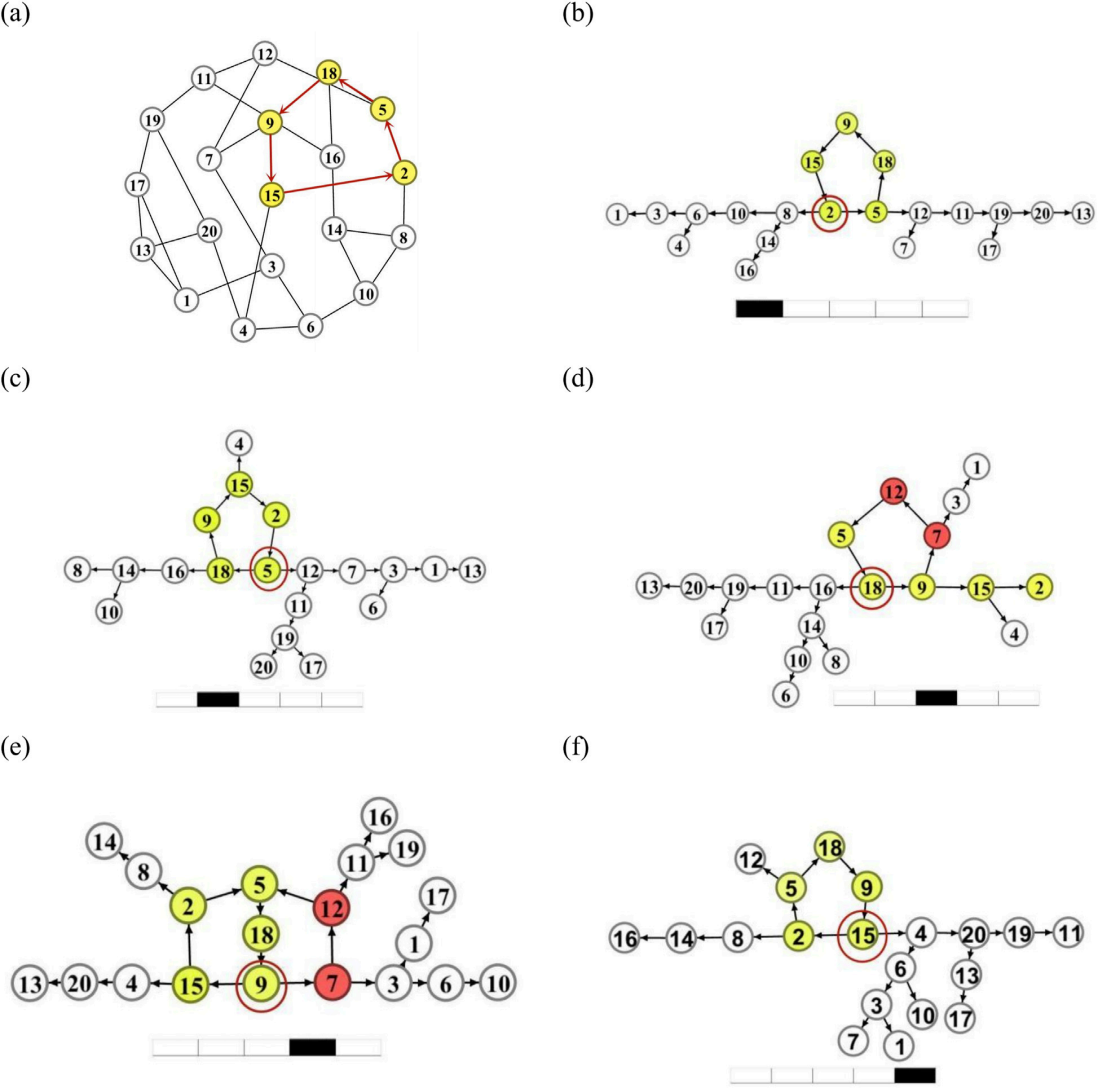
A test of the loop oscillation by starting from different nodes. **(a)**: The loop *L5* = (*2* →*5*→*18*→*9*→*15*→*2*) labeled as yellow nodes and red arrows on the network. **(b-f)**: The DPAD graphs of the network when the spike propagates to node *i* = *2* **(b)**, 5, **(c)**, 18 **(d)**, 9 **(e)**, and 15 **(f)** respectively on the loop L5.

We first focus on the stability of oscillation generated by an L=5 loop in a homogeneous random network presented in Figure 4a, which is labeled by yellow nodes and red links $\left(L_{5}=\left(2\to 5\to 18\to 9\to 15\to 2\right)\right)$ . It has been revealed that Qian et al. (2019) the minimum size of the Winfree loop for supporting stable oscillation is $L_{\min}=4$ . Therefore the L=5 loop is a short size.

We assume initially there exists a sustained oscillation on this loop. Moreover, we initially disconnect this loop with other neurons, which are set to be in the resting state. By connecting the oscillatory loop to the whole network at different instants, we monitor the consequent evolution of the whole network and check the long-time oscillation mode in terms of the DPAD method.

We first observe the long-term oscillatory pattern of the entire network by reconnecting other nodes when the pulse propagates from neuron i=1 to i=2 , which is presented in Figure 4b. It can be clearly found that the original loop $L_{5}$ is still the oscillation source, and other neurons play the role of propagators.

Similar behavior can be observed in Figures 4c,f, when the loop connects to other nodes as the pulse on the loop propagates to neurons i=5 and i=15 respectively. The oscillation source $L_{5}$ still keeps stable in these two cases. The difference between Figures 4b,c,f is the distribution of propagating paths.

In Figure 4d, we plot the long-term DPAD network pattern for the case when the pulse on the loop propagates to neuron i=18 and meanwhile the loop connects to other nodes. It can be found that a new loop with the same size $L=5\;L_{5}^{\prime }=\left(18\to 9\to 7\to 12\to 5\to 18\right)$ acts as the oscillation source by replacing the original loop. Two new neurons i=7,12 labeled in red join in the oscillation source, and neurons i=2 and i=15 become the propagators. This implies for this case the oscillation sustained by the original loop $L_{5}$ becomes unstable. 1nterestingly, as can be observed in Figure 4e when the pulse on the loop propagates to neuron i=9 and meanwhile the loop connects to other nodes, the original loop $L_{5}$ can coexist with the loop $L_{5}^{\prime }$ by sharing three common neurons i=5,18,9 . This forms an interesting double sources with two equal size Winfree loops. Moreover, it can be inferred that the source loop $L_{5}$ is neutrally stable due to the existence of a “mirror” loop.

The above numerical experiments indicate that the oscillation pattern sustained by a short loop is stable.

### 4.2 Stability of long-loop oscillation

**FIGURE 5:**
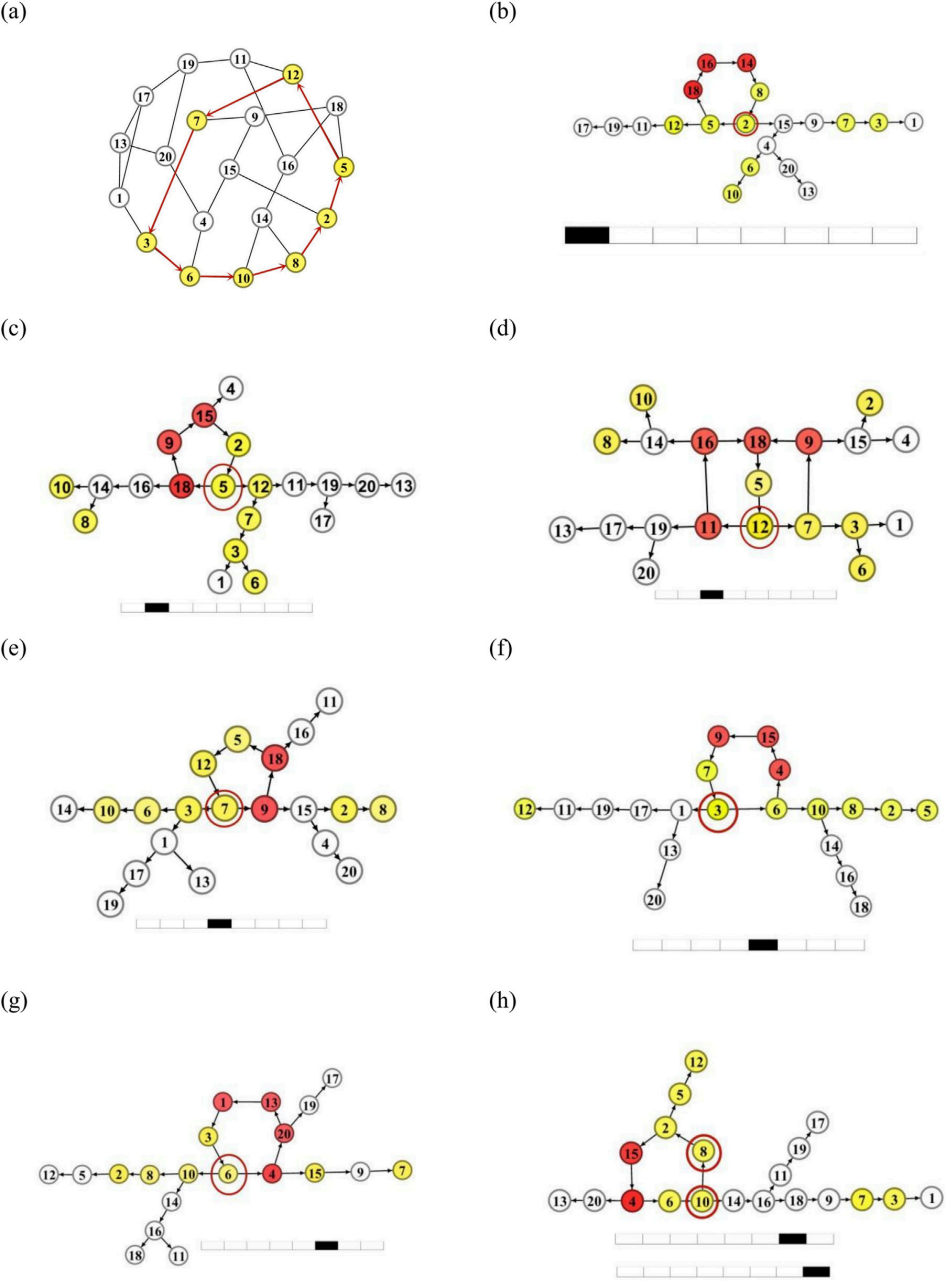
Test of the loop-oscillation stability for a loop with length *L* = *8*. **(a)**: The same network with a loop *L8* = (*2→5→12→7→3→6→10→8→2*) labeled as yellow nodes and red arrows on the network. **(b-h)**: The DPAD graphs of the network when the spike propagates to node *i* = *2* **(b)**, 5, **(c)**, 12 **(d)**, 7 **(e)**, 3 **(f)**, 6 **(g)**, 10 and 8 **(h)**, respectively on the loop L8.

Let us further examine the stability of oscillation supported by a longer loop. To do this, we choose a longer loop with L=8 neurons shown in Figure 5a, where the loop $L_{8}=\left(2\to 5\to 12\to 7\to 3\to 6\to 10\to 8\to 2\right)$ may support a longer-period oscillation. Similar to the above procedure, one first produces a sustained oscillation on this loop by disconnecting to other neurons. Then the loop is reconnected to other neurons according to the topology of Figure 5a at different instants.

In Figures 5b–h, the DPAD oscillation patterns of the network Figure 5a are presented by reconnecting the loop $L_{8}$ with other neurons as the spike propagates sequentially to i=2,5,12,7,3,6,10 and 8. One clearly finds that the sustained oscillation generated by a long loop $L_{8}$ is always unstable and replaced by oscillation sources with shorter loops. For example, loops with length L=5 will replace the $L_{8}$ loop and emerge as new oscillation sources in Figures 5c,d (two coexisting $L_{5}$ loops), and Figure 5e. One can also detect $L_{6}$ loops replace the long $L_{8}$ loop and perform as new oscillation sources in Figures 5b,f–h.

Therefore, one can conclude that a longer loop is more unstable and is easier to be replaced by shorter loops. As shown in Figures 5b–h, all new source loops share common neuron nodes with the unstable long loop. This indicates that these new shorter loops are neighbor loops of the long loop.

### 4.3 Loops on loop: Stability analysis

Oscillatory dynamics discussed above implies strong evidence of the competition among loops in the network. Obviously, the above results reveal that the stability of the oscillation mode on a given loop depends on its neighbor loops. A neighbor loop is excited only if the spike propagate through their common nodes or edges, the neighbor loop will also generate an oscillation mode and then compete with the original loop oscillation.

**FIGURE 6:**
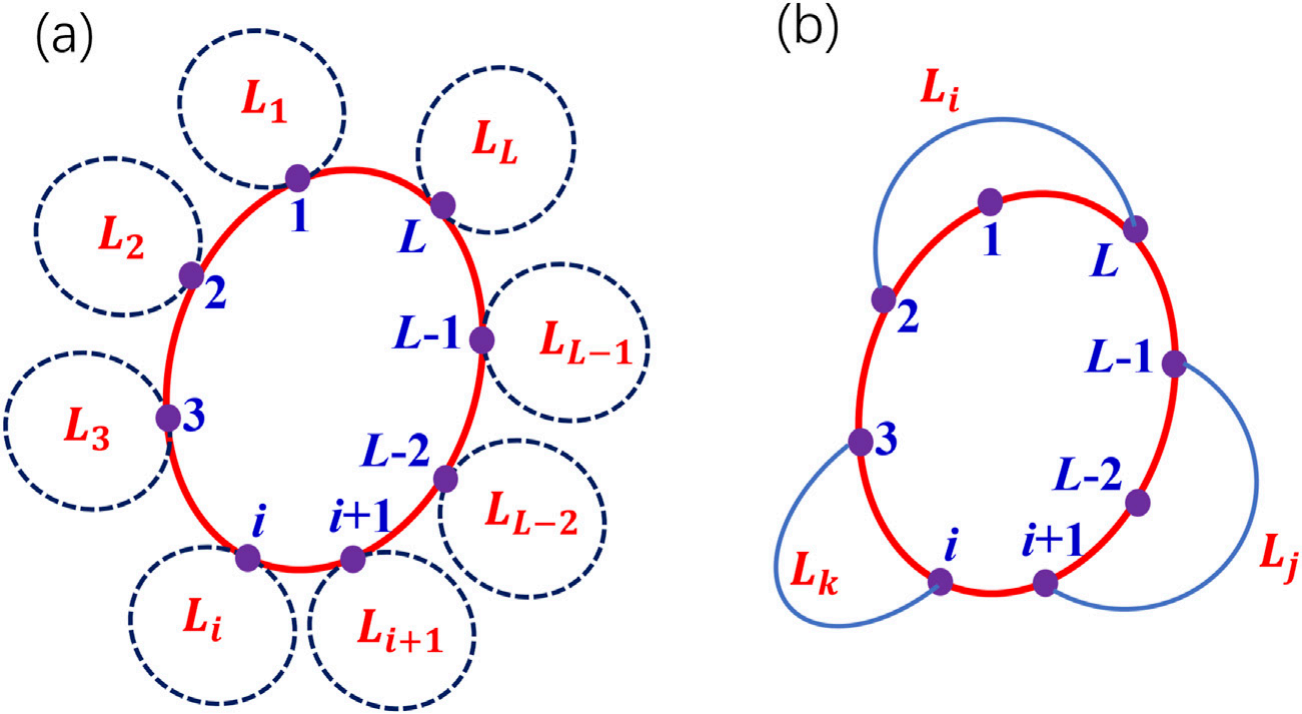
A schematic plot of the neighbor loops of a given loop on a network. **(a)** all the neighbor loops sharing one common node of a given loop. **(b)** Some neighbor loops sharing one common edge (two common nodes) or one common path (multiple common nodes).

In Figure 6, we schematically plot all possible neighbor loops of a given L -loop. The first level of neighbor loops contains those loops sharing one common node with L -loop, as shown in Figure 6a by $\left\{L_{i},\;i=1,2,\ldots ,L\right\}$ . The second level of neighbor loops includes all those loops sharing one common edge $e_{i,i+1}$ with two common nodes, e.g., $L_{k}$ in Figure 6b. Higher levels of neighbor loops sharing a common path $l_{i,i+m}=e_{i,i+1}e_{i+1,i+2}\ldots e_{i+m-1,i+m}$ with m+1 nodes, e.g., loops $L_{i}$ and $L_{j}$ in Figure 6b, can also be taken into account.

Usually the oscillation mode of a shorter loop has the advantage over that of a longer neighbor loop and dominate the oscillation on a network. For two non-neighboring loops, oscillation mode from the longer loop still persist if initially all neurons on the shorter loop are in resting states. This has been verified in Figure 4. Therefore, loops on loop determine the stability of the oscillation mode L . This means that an oscillation mode L is stable only if all its neighbor loops are longer than the L -loop, namely, $L_{k}>L$ , where k denotes all possible neighbor loops sharing common nodes, edges, and paths.

If there exists some k that $L_{k}<L$ , then the oscillation mode L becomes unstable as a spike passes these nodes, edges, or paths because this spike will excite neighboring shorter loops. In this case the oscillation mode L is *partially stable* (PS). This happens as long as the L loop is connected to the whole network when the spike propagates at those nodes or edges with shorter neighboring loops, for example, at one of nodes on the L -loop in Figure 6a, or at an edge or a path in 6(b). For these situations, the L oscillation mode will persist and dominate the oscillation and propagation on the whole network.

This stability analysis well explains the loop competitions observed in Figures 4, 5. The $L_{5}$ loop proposed in Figure 5 is so short that almost all its neighbor loops are longer than it, i.e., $L_{k}>L=5$ . Therefore one may observe its high stability in dominating the global oscillation. Nevertheless, for the longer loop L=8 proposed in Figure 5, the oscillation mode becomes unstable for almost all cases, which is replaced by the oscillation modes L=5 or L=6 .

## 5 Loop-hub competition and oscillation suppression

Topologically, loops describe the homogeneity property on a network, while hubs, i.e., nodes with large degrees, represent the heterogeneity. The above studies emphasize the role of loops and the impact of loop-loop competitions. It is also natural to explore the role of hubs in affecting the sustained oscillation generated by loops.

**FIGURE 7:**
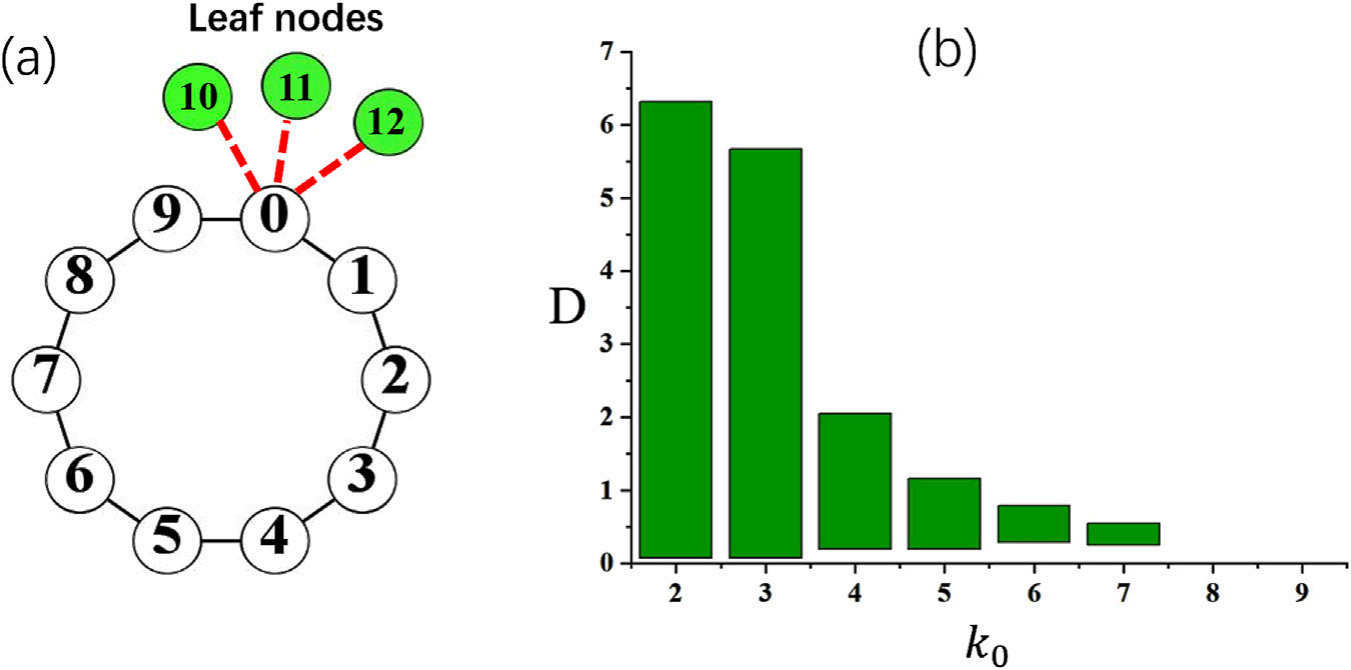
**(a)** A schematic plot of a loop with a heterogeneous hub node. In this example, a hub i=0 on a L=10 loop owns some leaves except for its two neighbors i=1,9 on the loop. **(b)** The sustained oscillation range of the coupling strength K for varying with the degree of the hub.

To do this, the simplest way is to design a hub on the loop. This idea is shown in Figure 7a, where one node, say, node i=0 , becomes a hub on the L loop by adding more leaves i=L,L+1,L+2,… . This results in the broken topological heterogeneity on a homogeneous loop network. We are concerned with the stability of oscillation propagation on the loop by modulating the degree of hub heterogeneity (the number of leaf nodes). We first generate a sustained oscillation as the propagation of spikes along the loop while node i=0 has no leaf nodes, namely, all nodes possess the same degree k=2 . Then one adds leaf nodes to check whether the oscillation keeps to be sustained.

In Figure 7b, the range of the coupling strength D for the sustained oscillation is plotted. It can be clearly found that the oscillation range shrinks rapidly with increasing the degree of the hub $k_{0}$ . The most drastic change occurs at $k_{0}=4$ , namely, two more leaf nodes are added to one of the nodes on the loop. When $k_{0}=8$ , it is hard to find a finite coupling interval in supporting oscillation on the loop. This tendency indicates that sustained oscillation is parametrically suppressed by increasing the degree of hub heterogeneity.

**FIGURE 8:**
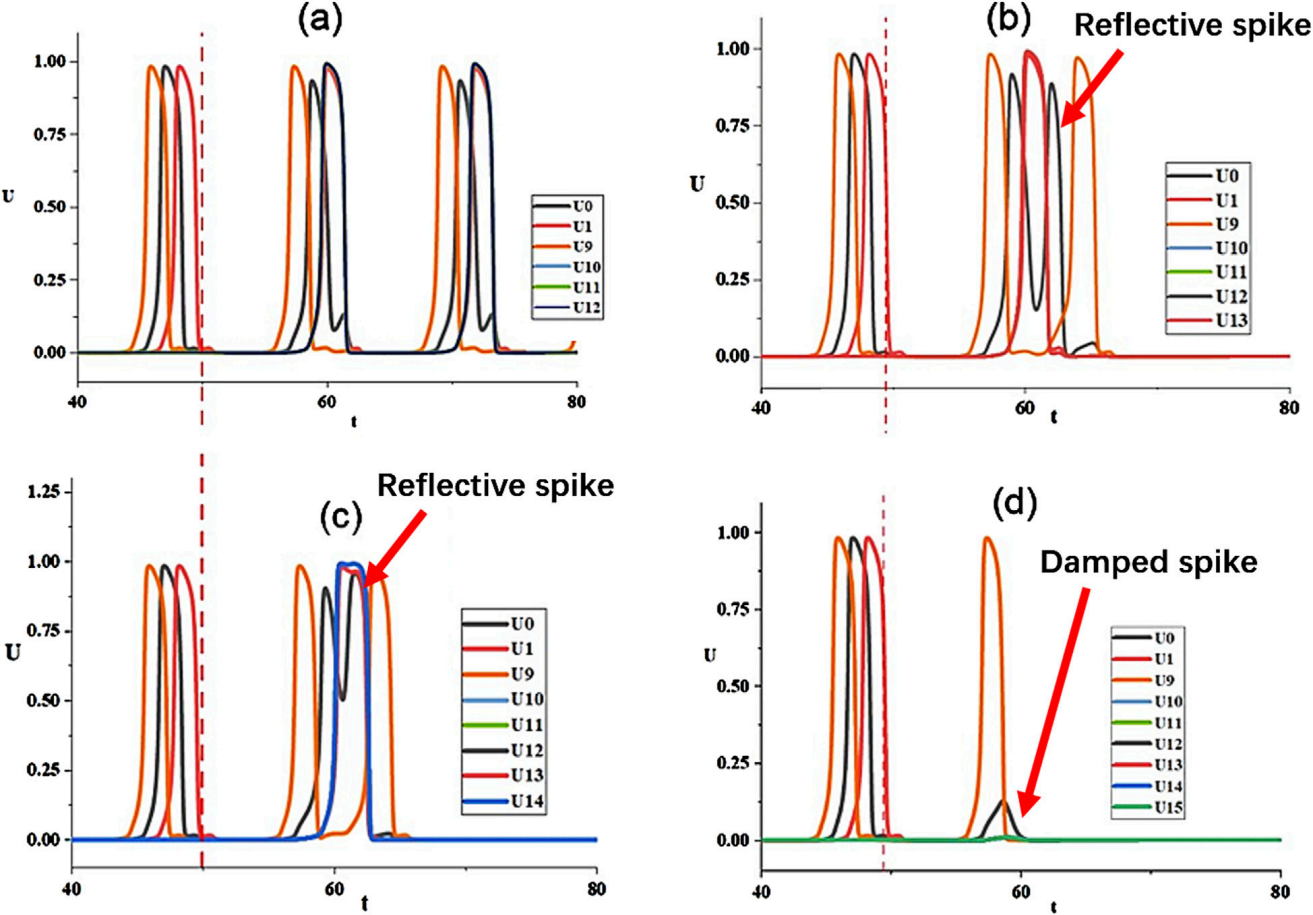
Evolution of $u_{i}\left(t\right)$ for different degrees of the hub node. Only the hub node (black line) $u_{0}\left(t\right)$ , its neighbor nodes $u_{1,9}\left(t\right)$ , and its leaf nodes $u_{i\geq 10}\left(t\right)$ are plotted. **(a)** $k_{0}=5$ ; **(b)** $k_{0}=6$ ; **(c)** $k_{0}=7$ ; **(d)** $k_{0}=8$ .

It is significant to unveil the physical mechanism of oscillation suppression of hub nodes. This is explored in Figure 8 by tracking the evolution of the hub node and its neighbor nodes, including neighbors on the loop and leaf nodes for the coupling strength K=1.0 . In Figure 8a, the spiking process of node i=0 , its two neighbors i=1,9 and its three leaves i=10,11,12 are plotted, where leaf nodes are linked at t=50.0 . One can find that spikes of i=1,0,9 follow the normal phase-advanced order, and the other three leaves are excited simultaneously.

The spiking process for the case of the hub i=0 with degree $k_{0}=6$ is presented in Figure 8b. As a comparison, as soon as the leaf nodes are switched on, the hub experiences a secondary spike closely followed by the first spike around t=60 . This secondary excitation is a reflective spike induced by the spikes at leaf nodes, forming an echo wave propagating back to the hub. The sustained oscillation on the loop disappears due to the emergence of this echo wave. Similar story occurs for the case of $k_{0}=7$ shown in Figure 8c. When $k_{0}$ becomes larger, as shown in Figure 8d for $k_{0}=8$ , the spike becomes damped and the oscillatory wave fails to propagate.

**FIGURE 9:**
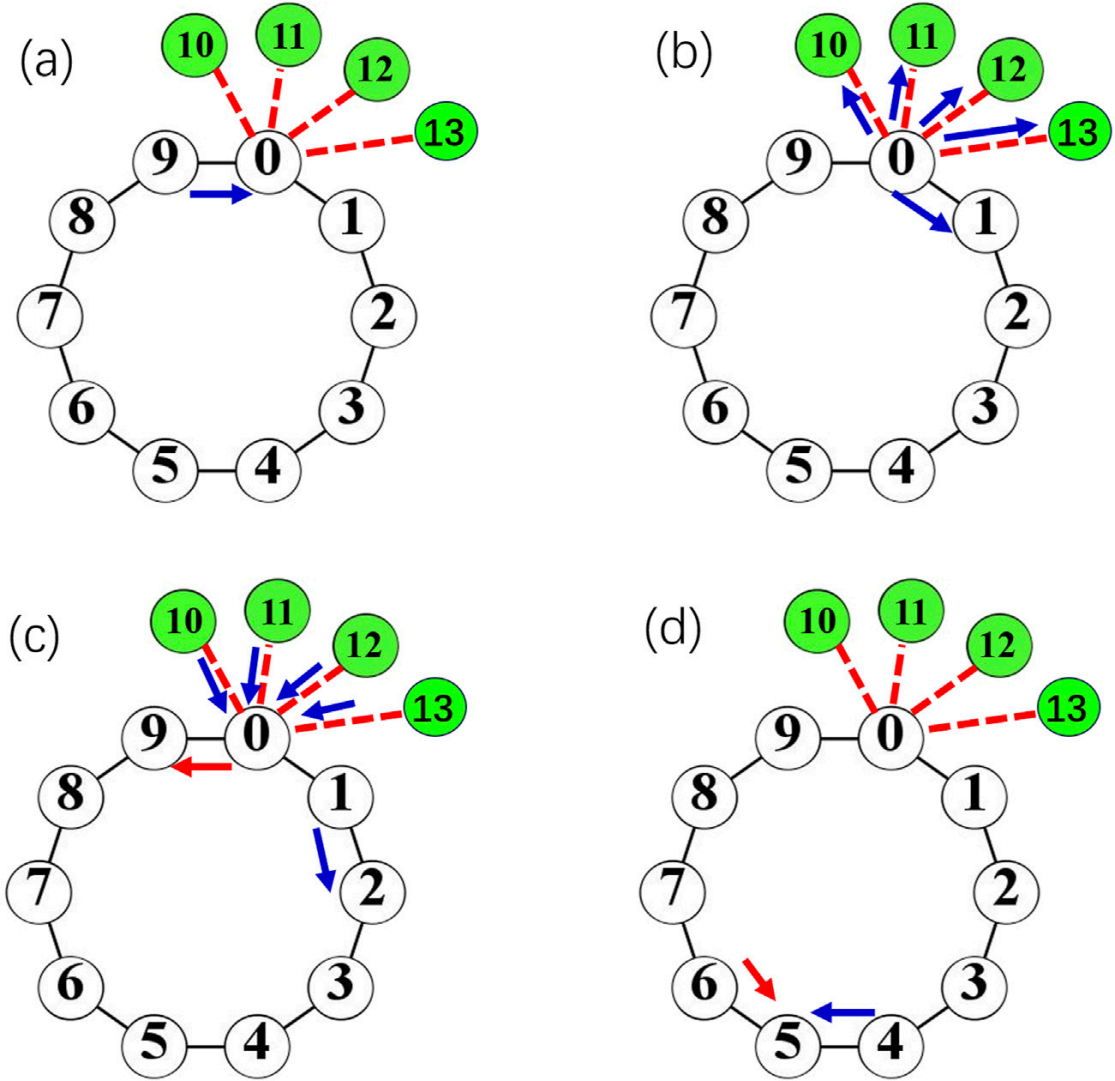
**(a -d)**: The process and the consequence of the generation of reflective spike labeled by red arrow for the large degree of the hub, e.g., k_0_ = 6. Nodes in green are leaves connected to the hub *i* = *0*. Blue arrow corresponds to a forward propagating spike wave front. The blue front propagates to the hub *i* = *0* **(a)**, and move forward along the loop and to the leaf nodes *i* = *10,11,12,13* **(b)**. The pulse is kicked back from leaf nodes and leads to a back-propagating wave along the loop (labeled as a red arrow) **(c)**, which collides with the forward wave (blue arrow) and annihilate **(d)**.

The process and the consequence of the generation of reflective spike are shown in Figure 9. Figure 9a shows the situation when a spike labeled as the blue arrow propagates clockwise along the loop. When the spike passes over the hub node i=0 , as shown in Figure 9b, leaf nodes i=10,11,12,13 are simultaneously excited and experience spikes. These spikes will conversely excite the hub shortly even though the hub does not relax back to the resting state, generating an anti-wave that propagates anti-clockwise along the loop, see the red arrow in Figure 9c. The anti-wave propagates reversely because the node i=9 is almost in resting state and easier to be excited as compared with the right-side node i=1 . This anti-wave propagates reversely and collides with the spiking wave propagating along the forward direction, as shown in Figure 9d. This collision results in the annihilation of the oscillation on the loop, as can be seen in Figure 8b.

The above analysis emphasize the important role of leaf nodes as a reflection wall, and the spike propagated to this “wall” will be bounced back and propagate in the reversed direction of the original spike wave. It can also be found that a critical number of leaves $n_{c}$ are necessary to exceed the spiking threshold of the hub. This wall also forms an obstacle that hinders the propagation of spiking wave on the loop. This is just the case observed in Figure 8d, where the spike is damped as the spike passes through the hub node. In this case, the sustained oscillation will be suppressed when the hub possesses more neighbors.

The above discussions is focused the effect of a single hub on a loop. The degree of the hub is shown to play a primary role in affecting and hindering the propagation on the loop. In fact, the scenario of hub effect can be naturally extended to networks with multiple hubs. Generally, the loop oscillation cannot be sustained on loops containing hubs. Therefore one has two consequences. First, sustained oscillation modes cannot be supported on a highly dense network, where most of the nodes possess very high degrees. In this case, sustained oscillation fails to occur on a homogeneous dense network with high average degree. Second, Winfree loops sustaining oscillations on networks are usually composed of those low-degree nodes. Suppose a heterogeneous network with distinct distributions of degrees, an effective Winfree that supports stable oscillation should avoid those nodes with large degrees. A representative example is the interesting long-period oscillation on scale-free networks, where long loops composed of low-degree nodes (Mi et al., 2013). This long-period oscillation mode is shown to be related to the long-term memory in the brain.

## 6 Concluding remarks

To summarize, in this paper, we extensively explored the competition dynamics of sustained oscillations on neuronal networks. Loops are identified as the basic building blocks of sustained oscillation. There exist a huge number of loops on a realistic network as potential candidates of the source in supporting the sustained oscillation, hence it is beneficial to explore competitive dynamics of these different oscillatory modes and determine the dominant loop governing the fundamental rhythm on the network.

As the first step, we briefly discussed multiple coexisting oscillatory patterns of networks of neurons with the same topologies and parameters. These different modes are dynamical attractors with different while complicated basins of attractions (Mi et al., 2011). These different patterns can be decomposed into the oscillation emerged from the source loop and the propagating spiking wave along different paths in terms of the DPAD approach.

The stability of the potential oscillation mode supported by a given loop can be checked by embedding it to the large network. It was found that a shorter loop possesses higher stability, i.e., it is difficult to compete a short-loop oscillatory mode by other potential long-loop modes. We further presented the loops-on-loop criteria in judging the global and partial stability of a given loop oscillation. The stability of the loop oscillation depends on the competition between the given loop and its surrounding neighbor loops.

Heterogeneity on networks may strongly affect the loop-supported oscillation. For simplicity, we focus on the case of a loop with one hub node linked with different leaf nodes. It is revealed that with increasing number of leaf nodes, the hub hinders the propagation of spiking wave, which highlights the important role of leaf nodes as a reflection wall, where the spiking wave is bounced back to form the anti-wave on the loop. A critical number of leaves $n_{c}$ is necessary to exceed the spiking threshold of the hub. In this case, the sustained oscillation will be suppressed when the hub possesses more neighbors. This implies stable oscillation loops with low-degree nodes are preferred.

Based on both loop-loop and loop-hub competitions, it can be inferred that an optimal stable loop that is capable of sustaining stable oscillation on complex networks should be short in length and low in node degree. This happens ubiquitously on homogeneous networks, where a large number of loops with various lengths can be found. For heterogeneous networks such as scale-free networks, sustainable loops should avoid those hub nodes, and hence some long loops get the opportunity in supporting sustained oscillations on networks.

There still are a number of challenges remained to be further studied. An important topic is the effect of transmission delay among neurons on the formation of Winfree loop and the propagation process in neuron networks.

Loop-sustained oscillations can be ubiquitously found in neuron networks, which implies a topology dominated emergence of functioning. Because they are related with various important oscillatory rhythms that are responsible for functions, we believe our results should be valuable in exploring the possible rhythmic behaviors and the corresponding functions such as memory in physiology of neural networks.

We contribute the present work to the memory of professor Hermann Haken for his contributions. Sustained oscillation on neuron networks is a typical dynamical self-organization behavior. There exist a large number of loops with distinct oscillatory modes. The competition among these modes and the emergence of dominant mode is the consequence of slaving principle proposed by Haken (Haken, 1983).

## Data Availability

The original contributions presented in the study are included in the article/supplementary material, further inquiries can be directed to the corresponding author.

## References

[B1] Arenas A. Díaz-Guilera A. Kurths J. Moreno Y. Zhou C. (2008). Complex networks: structure and dynamics. Phys. Rep. 469, 93–153. 10.1016/j.physrep.2005.10.009

[B2] Bär M. Eiswirth M. (1993). Turbulence due to spiral breakup in a continuous excitable medium. Phys. Rev. E 48, R1635–R1637. 10.1103/PhysRevE.48.R1635 9960866

[B3] Barabási A.-L. Oltvai Z. N. (2004). Network biology: understanding the cell’s functional organization. Nat. Rev. Genet. 5, 101–113. 10.1038/nrg1272 14735121

[B4] Bianconi G. Capocci A. (2003). Number of loops of size *h* in growing scale-free networks. Phys. Rev. Lett. 90, 078701. 10.1103/PhysRevLett.90.078701 12633275

[B5] Bullmore E. T. Sporns O. (2009). Complex brain networks: graph theoretical analysis of structural and functional systems. Nat. Rev. Neurosci. 10, 186–198. 10.1038/nrn2575 19190637

[B6] Buzsáki G. Draguhn A. (2004). Neuronal oscillations in cortical networks. Science 304, 1926–1929. 10.1126/science.1099745 15218136

[B7] Cross M. Greenside H. (2009). Pattern formation and dynamics in nonequilibrium systems. Cambridge: Cambridge University Press.

[B8] Cross M. C. Hohenberg P. C. (1993). Pattern formation outside of equilibrium. Rev. Mod. Phys. 65, 851–1112. 10.1103/RevModPhys.65.851

[B9] Fan T. Lu L. Shi D. Zhou T. (2020). Characterizing cycle structure in complex networks. Commun. Phys. 4, 272–279. 10.1038/s42005-021-00781-3

[B10] Fries P. (2005). A mechanism for cognitive dynamics: neuronal communication through neuronal coherence. Trends Cognitive Sci. 9, 474–480. 10.1016/j.tics.2005.08.011 16150631

[B11] Gutkin B. S. Laing C. R. Colby C. L. Chow C. C. Ermentrout G. B. (2001). Turning on and off with excitation: the role of spike-timing asynchrony and synchrony in sustained neural activity. J. Comput. Neurosci. 11, 121–134. 10.1023/A:1012837415096 11717529

[B12] Haken H. (1983). *Synergetics: an introduction*, vol. 1 of *springer series in synergetics* . 3rd rev. and enl. Berlin: Springer. 10.1007/978-3-642-88338-5

[B13] Hopfield J. J. (1982). Neural networks and physical systems with emergent collective computational abilities. Proc. Natl. Acad. Sci. 79, 2554–2558. 10.1073/pnas.79.8.2554 6953413 PMC346238

[B14] Hopfield J. J. (1984). Neurons with graded response have collective computational properties like those of two-state neurons. Proc. Natl. Acad. Sci. 81, 3088–3092. 10.1073/pnas.81.10.3088 6587342 PMC345226

[B15] Izhikevich E. M. (2006). Dynamical systems in neuroscience: the geometry of excitability and bursting. The MIT Press. 10.7551/mitpress/2526.001.0001

[B16] Jiang S. Zhou J. Small M. Lu J.-a. Zhang Y. (2023). Searching for key cycles in a complex network. Phys. Rev. Lett. 130, 187402. 10.1103/PhysRevLett.130.187402 37204881

[B17] Kim H.-J. Kim J. M. (2005). Cyclic topology in complex networks. Phys. Rev. E 72, 036109. 10.1103/PhysRevE.72.036109 16241517

[B18] Liao X. Xia Q. Qian Y. Zhang L. Hu G. Mi Y. (2011). Pattern formation in oscillatory complex networks consisting of excitable nodes. Phys. Rev. E 83, 056204. 10.1103/PhysRevE.83.056204 21728627

[B19] Marder E. Bucher D. (2001). Central pattern generators and the control of rhythmic movements. Curr. Biol. 11, R986–R996. 10.1016/S0960-9822(01)00581-4 11728329

[B20] Marder E. Bucher D. Schulz D. J. Taylor A. L. (2005). Invertebrate central pattern generation moves along. Curr. Biol. 15, R685–R699. 10.1016/j.cub.2005.08.022 16139202

[B21] Marinari E. Semerjian G. Van Kerrebroeck V. (2007). Finding long cycles in graphs. Phys. Rev. E 75, 066708. 10.1103/PhysRevE.75.066708 17677390

[B22] Mcintosh A. R. (2000). Towards a network theory of cognition. Neural Netw. official J. Int. Neural Netw. Soc. 13 (8-9), 861–870. 10.1016/s0893-6080(00)00059-9 11156197

[B23] Mi Y. Liao X. Huang X. Zhang L. Gu W. Hu G. (2013). Long-period rhythmic synchronous firing in a scale-free network. Proc. Natl. Acad. Sci. 110. E4931, E4936. 10.1073/pnas.1304680110 24277831 PMC3864271

[B24] Mi Y. Zhang L. Huang X. Qian Y. Hu G. Liao X. (2011). Complex networks with large numbers of labelable attractors. Europhys. Lett. 95, 58001. 10.1209/0295-5075/95/58001

[B25] Newman M. E. (2003). The structure and function of complex networks. SIAM Rev. 45, 167–256. 10.1137/s003614450342480

[B26] Noh J. D. (2007). Loop statistics in complex networks. Eur. Phys. J. B 66, 251–257. 10.1140/epjb/e2008-00401-9

[B27] Othmer H. G. Scriven L. E. (1971). Instability and dynamic pattern in cellular networks. J. Theor. Biol. 32, 507–537. 10.1016/0022-5193(71)90154-8 5571122

[B28] Othmer H. G. Scriven L. E. (1974). Non-linear aspects of dynamic pattern in cellular networks. J. Theor. Biol. 43, 83–112. 10.1016/s0022-5193(74)80047-0 4813541

[B29] Park H.-J. Friston K. (2013). Structural and functional brain networks: from connections to cognition. Science 342, 1238411. 10.1126/science.1238411 24179229

[B30] Pikovsky A. Rosenblum M. Kurths J. (2001). Synchronization: a universal concept in nonlinear sciences. Cambridge: Cambridge University Press.

[B31] Qian Y. Cui X. Zheng Z. (2017). Minimum winfree loop determines self-sustained oscillations in excitable erdös-rényi random networks. Sci. Rep. 7, 5746. 10.1038/s41598-017-06066-6 28720831 PMC5516026

[B32] Qian Y. Huang X. Hu G. Liao X. (2010). Structure and control of self-sustained target waves in excitable small-world networks. Phys. Rev. E 81, 036101. 10.1103/PhysRevE.81.036101 20365809

[B33] Qian Y. Zhang G. Wang Y. Yao C. Zheng Z. (2019). Winfree loop sustained oscillation in two-dimensional excitable lattices: prediction and realization. Chaos An Interdiscip. J. Nonlinear Sci. 29, 073106. 10.1063/1.5085644 31370411

[B34] Rabinovich M. I. Varona P. Selverston A. I. Abarbanel H. D. I. (2006). Dynamical principles in neuroscience. Rev. Mod. Phys. 78, 1213–1265. 10.1103/RevModPhys.78.1213

[B35] Rodrigues F. A. Peron T. K. D. M. Ji P. Kurths J. (2016). The kuramoto model in complex networks. Phys. Rep. 610, 1–98. 10.1016/j.physrep.2015.10.008

[B36] Roxin A. Riecke H. Solla S. A. (2004). Self-sustained activity in a small-world network of excitable neurons. Phys. Rev. Lett. 92, 198101. 10.1103/PhysRevLett.92.198101 15169447

[B37] Seth A. K. Bayne T. (2022). Theories of consciousness. Nat. Rev. Neurosci. 23, 439–452. 10.1038/s41583-022-00587-4 35505255

[B38] Singer W. (1999). Neuronal synchrony: a versatile code for the definition of relations? Neuron 24, 49–125. 10.1016/s0896-6273(00)80821-1 10677026

[B39] Steele A. J. Tinsley M. Showalter K. (2006). Spatiotemporal dynamics of networks of excitable nodes. Chaos An Interdiscip. J. Nonlinear Sci. 16, 015110. 10.1063/1.2177569 16599776

[B40] Strogatz S. (1994). Nonlinear dynamics and Chaos : with applications to physics biology chemistry and engineering. Reading, Mass. Addison-Wesley Pub.

[B41] Tiesinga P. Sejnowski T. J. (2009). Cortical enlightenment: are attentional gamma oscillations driven by ing or ping? Neuron 63, 727–732. 10.1016/j.neuron.2009.09.009 19778503 PMC2778762

[B42] Tinsley M. Cui J. Chirila F. V. Taylor A. Zhong S. Showalter K. (2005). Spatiotemporal networks in addressable excitable media. Phys. Rev. Lett. 95, 038306. 10.1103/PhysRevLett.95.038306 16090780

[B43] Turing A. M. (1952). The chemical basis of morphogenesis. Philosophical Trans. R. Soc. Lond. Ser. B, Biol. Sci. 237, 37–72. 10.2307/92463 PMC4360114 25750229

[B44] Wang X.-J. (2010). Neurophysiological and computational principles of cortical rhythms in cognition. Physiol. Rev. 90, 1195–1268. 10.1152/physrev.00035.2008 20664082 PMC2923921

[B45] Wilson H. R. Cowan J. D. (1972). Excitatory and inhibitory interactions in localized populations of model neurons. Biophysical J. 12, 1–24. 10.1016/S0006-3495(72)86068-5 4332108 PMC1484078

[B46] Winfree A. T. (1967). Biological rhythms and the behavior of populations of coupled oscillators. J. Theor. Biol. 16, 15–42. 10.1016/0022-5193(67)90051-3 6035757

[B47] Winfree A. T. (1991). Varieties of spiral wave behavior: an experimentalist’s approach to the theory of excitable media. Chaos An Interdiscip. J. Nonlinear Sci. 1, 303–334. 10.1063/1.165844 12779929

[B48] Zhang Z. Huang X. Zheng Z. (2014a). Exploring cores and skeletons in oscillatory gene regulatory networks by a functional weight approach. Sci. China Phys. Mech. & Astronomy 57, 1319–1333. 10.1360/SSPMA2014-00123

[B49] Zhang Z. Li Z. Qian Y. Hu G. Zheng Z. (2014b). Exploring cores and skeletons in oscillatory gene regulatory networks by a functional weight approach. Europhys. Lett. 105, 18003. 10.1209/0295-5075/105/18003

[B50] Zhang Z. Ye W. Qian Y. Zheng Z. Huang X. Hu G. (2012). Chaotic motifs in gene regulatory networks. PLoS ONE 7, e39355. 10.1371/journal.pone.0039355 22792171 PMC3391214

[B51] Zheng Z. (2004). Spatiotemporal dynamics and cooperative behavior of coupled nonlinear systems. Beijing: Higher Education Press (in Chinese).

[B52] Zheng Z. (2019). “Emergent dynamics of complex systems: from synchronization to collective transport,”. Beijing: Science Press (in Chinese).

[B53] Zheng Z. Cross M. C. (2003). Defect-induced propagation in excitable media. Int. J. Bifurcation Chaos 13, 3125–3133. 10.1142/s0218127403008491

[B54] Zheng Z. Qian Y. (2018). Dominant phase-advanced driving analysis of self-sustained oscillations in biological networks. Chin. Phys. B 27, 018901. 10.1088/1674-1056/27/1/018901

